# The Effect of Q Angle and Hamstring Length on Balance Performance in Gonarthrosis Patients

**DOI:** 10.7759/cureus.43615

**Published:** 2023-08-17

**Authors:** Rukiye Ciftci, Ahmet KURTOĞLU

**Affiliations:** 1 Department of Anatomy, Faculty of Medicine, Gaziantep Islami Bilim ve Teknoloji University, Gaziantep, TUR; 2 Department of Coaching, Faculty of Sports Sciences, Bandırma Onyedi Eylül University, Balıkesir, TKM

**Keywords:** control group, balance, hamstring length, q angle, gonarthrosis

## Abstract

Introduction

Gonarthrosis (G) is a progressive disease that affects the knee joint and causes pain and limitation of movement in the knee. The determination of the morphometric status of G patients has been a subject of interest recently. The purpose of this study is to determine how hamstring length (HL) and patellofemoral angle (Q angle [QA]) affect the ability of individuals with G to maintain balance.

Methods

A total of 80 (40 G patients and 40 in the control group [CG]) participants aged 40-65 were included in the study. A goniometer was used to measure the participants' QA. The mean age of the participants was 50.18±7.16 in the G group and 51.40±6.64 in CG. HL was measured using the sit-and-reach test. Participants' dynamic balance state was evaluated using the Y balance test by stepping in the following directions: right-anterior (R-An), right-posteromedial (R-Pm), right-posterolateral (R-Pl), left-anterior (L-An), left-posteromedial (L-Pm), and left-posterolateral (L-Pl).

Results

According to the results of this research, the balance performance of G patients was significantly lower in all directions (R-An, R-Pm, R-Pl, L-An, L-Pm, L-Pl) compared to the CG. In both groups, R-QA, L-QA, and HL did not change. However, linear regression analysis indicated that in G patients, R-QA, L-QA, and HL affected balance performance. No significant interaction was found between these parameters and balance performance in the CG.

Conclusion

HL and QA have a significant role in determining body balance. In our study, we found that QA decreased in G patients, leading to genu varum in these individuals. The hamstring muscle shortening observed in G patients significantly negatively affected balance, especially in the R-An, R-Pm, L-An, L-Pm, and L-Pl directions. For healthcare professionals preparing treatment protocols for G patients, we recommend providing exercises to improve balance, especially in these directions.

## Introduction

Osteoarthritis is the most common disease affecting the synovial joints and most commonly affecting the knee joint. Knee osteoarthritis is called gonarthrosis (G) [1,2]. In individuals over the age of 65, radiographic findings accompany the condition in 80% of cases, and one-third of those cases are symptomatic [3]. According to data from the World Health Organization, G is the fourth most common cause of disability in women and the eighth most common cause in men [4]. It is the most common pathology in G patients, especially in elderly individuals. Genu varum deformity (GVD) due to pain and loss of strength is common in these individuals [1]. The most practical way to determine GVD is by measuring the patellofemoral angle (Q angle [QA]) [5].

QA is commonly used in the kinematic evaluation of the knee joint and lower extremity [6]. QA is the angle of the quadriceps femoris muscle and is defined as the acute angle where the midline of the patella intersects with the line from the anterior superior of the spina iliaca anterior superior to the midpoint of the patella medially and laterally with the tibial tuberosity. This angle is known to have an effect on patellar translation [6,7]. It is believed that when QA is above 15-20 degrees, it impairs the extension mechanism of the knee joint and increases the tendency of the patella to shift laterally, leading to femoral pain [8]. Abnormally low values of QA have been emphasized to cause various pains and injuries [7].

The ideal way to choose a treatment plan for G patients is to evaluate their postural characteristics. Finding the physical and physiological traits that interact with one another is crucial for understanding treatment and enhancing performance in G patients [9]. Coronal tibial inclination has been identified as an important risk factor, especially in G patients with varus deformity [10]. The posterior cruciate ligament relaxes when the knee is flexed, increasing varus-valgus laxity and range of motion in the knee [11].

The shortening of the hamstring muscles makes a significant contribution to the occurrence of the G. While hamstring muscle tension increases the possibility of injury to the musculoskeletal system, it also prepares the ground for the formation of G. Along with diseases including patella femoral pain syndrome [12], patellar tendinitis [13], and lumbar disc herniation [14], short hamstrings are thought to be a major contributing factor in G. Short hamstrings can affect a person's ability to balance and their quality of life [15]. The impact of hamstring tightness on the biomechanics of G patients and how the measurement of QA, a clinical sign of G, affects people's balance in these pathologies remain unclear despite the literature's abundance of papers on the diagnosis and treatment of G patients.

## Materials and methods

To determine the sample size for the study, a power analysis was conducted using the G-Power program, with a type I error (α) of 0.05, a power (1-β) of 0.80, and an effect size of 1.3 [16]. As a result of the analysis, it was determined that there should be 36 participants for each group, 40 in the control group (CG) and 40 G patients, in the research. The study included individuals between 40 and 65 years of age who had not undergone any surgical procedures in the knee region and had no lower extremity amputations. Individuals who had undergone knee surgery or had knee implants or had mental health issues were excluded from the study. The study included patients diagnosed with G and treated in Bandırma Onyedi Eylül University Physical Medicine and Rehabilitation Service and individuals who were treated in the same service and did not have any pathology in their lower extremities.

G patients included in the study were diagnosed with G stage 3 and stage 4 based on the Kellgren-Lawrence classification and were recommended for surgery but refrained from undergoing the procedure due to various reasons (presence of a pandemic, fear of surgery, etc.) [3].

Ethical aspect of the study

The study was conducted with the approval of the Bandirma Onyedi Eylul University Non-Interventional Ethics Committee on June 21, 2023 (protocol number 2023/6). The study was carried out in accordance with the principles of the Helsinki Declaration, and each participant provided voluntary informed consent by signing a consent form.

Study design

After recording the participants' sociodemographic information, QA was measured using a goniometer and scaled in degrees. The sit-and-reach test was used to determine hamstring length (HL) and recorded in centimeters (cm). The Y balance test was used to evaluate the balance status of the participants and was scaled as %.

The ages of the participants were calculated in years. A barefoot steel stadiometer was used to measure their height and was recorded in centimeters. Their weight was measured in kilograms while standing without shoes using a Tanita BC Segmental Body Analysis System (Tanita Corporation, Tokyo, Japan).

Baseline® device (Cooper Institute/YMCA, AAHPERD [American Alliance for Health, Physical Education, Recreation and Dance], Baltimore, MD, USA) was used for the test. Before the measurement, the individuals were asked to sit with their heels placed on the testing device and warmed up by reaching forward three times. Then, the individuals’ arm length was determined on the device, and they were asked to reach forward as far as they could with their fingertips pushing the measurement apparatus without lifting their knees. Measurements were taken three times, and the average was recorded [17].

Evaluation of QA

The subject was lying supine on a flat examination table when the QA was taken, and both lower limbs were fully extended with the quadriceps muscle relaxed. The right knee was used to take the measurement. The measurements were taken using a computerized goniometer of the Lafayette brand. The anterior superior iliac spine, the center of the patella, and the tibial tubercle all had markers applied to them. The middle of the patella was in line with the midpoint of the goniometer. The arm of the goniometer was aligned with the anterior superior iliac spine point, and the other arm was aligned with the tibial tubercle point, and the QA was measured in degrees [18].

Y balance test

Balance was evaluated using the Y balance test. Participants were asked to stand on one foot at the center of the testing device and touch their toe to the anterior, posteromedial, and posterolateral directions while maintaining balance with the other foot. Hands were placed on the hips, and full contact with the supporting surface was ensured. After six attempts in each direction for both legs, the tests were repeated three times. Patients were asked to perform the Y balance test by stepping in the following directions: right-anterior (R-An), right-posteromedial (R-Pm), right-posterolateral (R-Pl), left-anterior (L-An), left-posteromedial (L-Pm), and left-posterolateral (L-Pl). The average of each direction was calculated and recorded in centimeters relative to leg length [19].

Statistical analysis

SPSS Version 25 (IBM Corp., Armonk, NY, USA) was used for statistical analysis in this study. The normality analysis of the data in the study was determined according to the Kolmogorov-Smirnov test, and it was determined that the data were normally distributed. In addition, Levene's test was applied for homogeneity of variances. As a result, the independent sample t-test was applied for statistical operations between CG and G groups. Linear regression analysis was applied to determine the relationship between QA and HL and balance performance indicators. In the study, the effect sizes were determined according to Cohen's d formula and according to the following formula: 0.2 means low effect, 0.5 means medium size, and 0.8 and above means high effect size. The level of significance in the study was determined as 0.05.

## Results

Upon examining the demographic data of the study, there was no statistically significant difference between the mean age of the CG (51.40±6.64) and the mean age of the G patients (50.18±7.16) (p=0.493). Similarly, there was no significant difference in the mean weight between the CG (70.33±18.05) (kg) and the G patients (69.31±15.01) (kg) (p=0.809). However, the mean height of the CG (170.86±9.91) (cm) was significantly higher compared to the mean height of the G patients (160.09±6.24) (cm) (p<0.001). Additionally, there was a statistically significant difference favoring the CG when comparing the mean BMI of G patients (26.92±5.14) (kg/m^2^) to the mean BMI of the CG (23.77±4.21) (kg/m^2^) (p=0.011) (Table 1).

**Table 1:** Demographic information of participants BMI, body mass index; CG, control group; G, gonarthrosis **p < 0.001; ***p < 0.01

Parameters	CG, mean ± SD	G, Mean ± SD	p-value
Age (year)	51.40±6.64	50.18±7.16	0.493
Weight (kg)	70.33±18.05	69.31±15.01	0.809
Height (cm)	170.86±9.91	160.09±6.24	<0.001**
BMI (kg/m^2^)	23.77±4.21	26.92±5.14	0.011***

**Figure 1:**
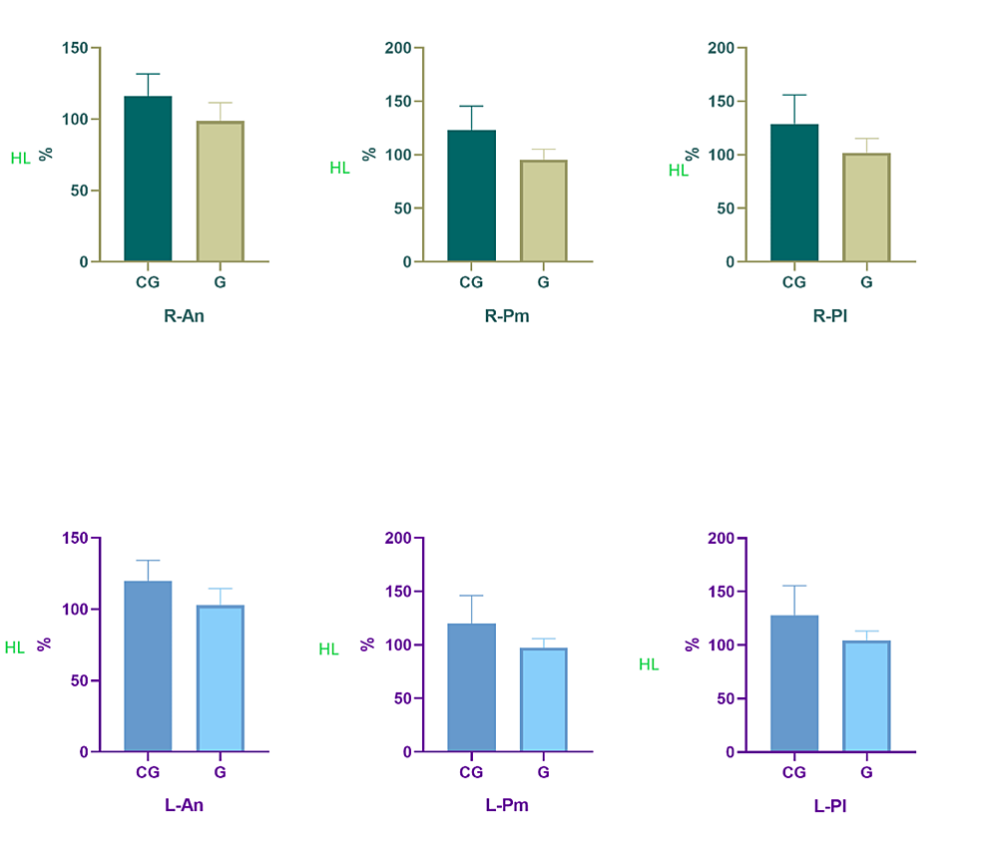
Comparison of participants' Q angles, hamstring lengths, and Y balance test performances CG, control group; G, gonarthrosis; HL, hamstring length; R-An, right-anterior; R-Pm, right-posteromedial; R-Pl, right-posterolateral; L-An, left-anterior; L-Pm, left-posteromedial; L-Pl, left posterolateral

In Table 2, the results of QA, HL, and Y balance test of the participants were compared. According to the results, there was no significant difference between G and CG in terms of R-QA, L-QA, and HL (p>0.05). However, in G patients, R-An (t=4.841, p<0.001, Cohen's d=1.23), R-Pm (t=6.384, p<0.001, Cohen's d=1.62), R-Pl (t=5.046, p<0.001, Cohen's d=1.28), L-An (t=5.077, p<0.001, Cohen's d=1.29), L-Pm (t=4.652, p<0.001, Cohen's d=1.18), and L-Pl (t=4.613, p<0.001, Cohen's d=1.17) values were significantly lower compared to the CG (Figure 1 and Table 2).

**Table 2:** Comparison of participants' Q angles, hamstring lengths, and Y balance test performances G, gonarthrosis; CG, control group; R-QA, right Q angle; L-QA, left Q angle; HL, hamstring length; R-An, right-anterior; R-Pm, right-posteromedial; R-Pl, right-posterolateral; L-An, left-anterior; L-Pm, left-posteromedial; L-Pl, left posterolateral **p < 0.01

Parameters	Group	N	Mean ± SD	t	p-value	Cohen’s d	95% CI	
							Lower	Upper
R-QA (angle)	CG	40	10.467±1.38	1.742	0.087	0.44	-0.063	0.945
	G	40	9.906±1.14					
L-QA (angle)	CG	40	10.467±1.38	1.457	0.150	0.37	-0.134	0.871
	G	40	9.969±1.30					
HL (cm)	CG	40	-5.400±10.01	-0.966	0.338	-0.24	-0.744	0.256
	G	40	-3.281±7.10					
R-An (%)	CG	40	116.144±15.59	4.841	<0.001**	1.23	0.682	1.770
	G	40	98.660±12.78					
R-Pm (%)	CG	40	123.063±22.29	6.384	<0.001**	1.62	1.041	2.193
	G	40	95.498±9.67					
R-Pl (%)	CG	40	128.969±26.98	5.046	<0.001**	1.28	0.730	1.826
	G	40	101.995±13.23					
L-An (%)	CG	40	119.712±14.49	5.077	<0.001**	1.29	0.737	1.835
	G	40	102.782±11.69					
L-Pm (%)	CG	40	119.970±26.06	4.652	<0.001**	1.18	0.637	1.719
	G	40	97.378±8.43					
L-Pl (%)	CG	40	127.925±27.51	4.613	<0.001**	1.17	0.628	1.709
	G	40	104.336±8.69					

In Table 3, the linear regression results between R-QA, L-QA, and HL of G patients and balance performance parameters were analyzed. According to the results, there was a significant and high-level relationship between R-QA results of G patients and R-An (t=6.177, p<0.001, 95% CI: 0.172 to 0.344), R-Pm (t=-3.512, p=0.002, 95% CI: -0.190 to -0.049), R-Pl (t=-4.980, p<0.001, 95% CI: -0.138 to -0.057), L-An (t=-5.932, p<0.001, 95% CI: -0.331 to -0.161), L-Pm (t=6.319, p<0.001, 95% CI: 0.206 to 0.405), and L-Pl (t=-5.184, p<0.001, 95% CI: -0.308 to -0.133). Similarly, G patients' L-QA results were significantly and highly correlated with R-An (t=7.165, p<0.001, 95% CI: .234 to .423), R-Pm (t=-2.983, p=0.006, 95% CI: -0.188 to -0.035), R-Pl (t=-4.882, p<0.001, 95% CI: -0.149 to -0.061), L-An (t=-7.275, p<0.001, 95% CI: -0.425 to -0.237), L-Pm (t=6.743, p<0.001, 95% CI: .249 to .468), and L-Pl (t=-6.557, p<0.001, 95% CI: -0.403 to -0.210). Furthermore, G patients' HL results showed a significant and high-level relationship with R-An (t=-4.113, p<0.001, 95% CI: -1.889 to -0.628), R-Pm (t=4.899, p=0.006, 95% CI: .708 to 1.734), L-An (t=2.095, p=0.046, 95% CI: .011 to 1.261), L-Pm (t=-4.667, p<0.001, 95% CI: -2.383 to -0.924), and L-Pl (t=5.641, p<0.001, 95% CI: 1.116 to 2.399) (Table 3).

**Table 3:** Comparison of linear regression analysis results between balance performance with R-QA, L-QA, and HL of patients with gonarthrosis R-QA, right Q angle; L-QA, left Q angle; HL, hamstring length; R-An, right-anterior; R-Pm, right-posteromedial; R-Pl, right-posterolateral; L-An, left-anterior; L-Pm, left-posteromedial; L-Pl, left-posterolateral *p < 0.05. **p < 0.006

Dependent variables	Predictors	Coefficients							R^2^
		B	SE	β	t	p-value	95% CI		
							Lower	Upper	
R-QA									
	CONSTANT	24.312	3.058		7.951		18.014	30.609	0.758
	R-An	-0.258	0.042	2.880	6.177		0.172	0.344	
	R-Pm	-0.119	0.034	-1.009	-3.512	0.002*	-0.190	-0.049	
	R-Pl	-0.098	0.020	-1.127	-4.980	0.001*	-0.138	-0.057	
	L-An	-0.246	0.041	-2.509	-5.932		-0.331	-0.161	
	L-Pm	0.306	0.048	2.249	6.319		0.206	0.405	
	L-Pl	-0.221	0.043	-1.672	-5.184		-0.308	-0.133	
L-QA	CONSTANT	29.997	3.358		8.932		23.080	36.913	0.776
	R-An	0.329	0.046	3.216	7.165		0.234	0.423	
	R-Pm	-0.111	0.037	-0.826	-2.983	0.006**	-0.188	-0.035	
	R-Pl	-0.105	0.022	-1.064	-4.882		-0.149	-0.061	
	L-An	-0.331	0.046	-2.963	-7.275		-0.425	-0.237	
	L-Pm	0.358	0.053	2.311	6.743		0.249	0.468	
	L-Pl	-0.306	0.047	-2.037	-6.557		-0.403	-0.210	
HL	CONSTANT	-104.288	22.391		-4.658		-150.404	-58.173	0.662
	R-An	-1.259	0.306	-2.265	-4.113		-1.889	-0.628	
	R-Pm	1.221	0.249	1.663	4.899		0.708	1.734	
	R-Pl	0.205	0.144	0.382	1.429	0.165	-0.091	0.501	
	L-An	0.636	0.304	1.047	2.095	0.046*	0.011	1.261	
	L-Pm	-1.653	0.354	-1.962	-4.667		-2.383	-0.924	
	L-Pl	1.757	0.311	2.150	5.641		1.116	2.399	

## Discussion

It was discovered in this study that the balance performance of G patients was considerably worse in all directions (R-An, R-Pm, R-Pl, L-An, L-Pm, L-Pl) compared to the CG when hamstring tightness and QA measurement were assessed. R-QA, L-QA, and HL did not significantly differ between the two groups, though. However, it was found that R-QA, L-QA, and HL significantly influenced balance performance in G patients based on the findings of the linear regression analysis, whereas no such interaction was seen in the CG.

Obesity and being overweight are considered important risk factors, especially in G patients. The results of a study in the literature showed a significant reduction in the risk of developing symptomatic and radiographic G in women who lost weight [20]. In our study, the BMI measurements of G patients were significantly higher. We believe that excessive weight increases the load on the lower extremities in G patients, leading to hamstring tightness and degeneration of the knee joint. The lack of mobilization in patients with G may cause these individuals to gain weight. For this reason, it is thought that there is a need for randomized controlled studies examining the causes of physical inactivity in patients with G [21].

An increase in QA in the knee leads to genu varum, while a decrease in QA leads to genu valgum. Tension is formed medially in the knee in the genu varum and laterally in genu valgum. In both cases, the body's center of gravity is altered, resulting in increased load on the lower extremities, especially the knee [22]. It has been reported that G patients tend to have genu varum. The reason for this is suggested to be the weakening of the vastus lateralis part of the medial quadriceps femoris and the tightness in the hamstring muscles in G patients [23,24]. In our study, although the QA angle in patients with G did not differ from that of CG, it was found to be lower on average. It is thought that the most important factor in the formation of this situation is due to the low mean age of the patients with G, who constitute the population of our study. In a recent study, it was concluded that the deterioration of QA in female G patients is frequently seen after the age of 60 [25]. For this reason, we think that the results of our research will contribute to the literature on the early diagnosis of QA deterioration in women with G and when the rehabilitation process begins.

Although the exact cause of hamstring tightness is not fully understood, it is believed to be primarily due to disuse atrophy and lack of activation [25]. Thus, the significant reason for the strength deficit in G patients appears to be related to hamstring muscle strength [26,27]. In our study, although HL was shorter in G patients, it was not statistically significant. However, we concluded that HL directly influenced balance performance in G patients. İn the literature, no significant changes were observed in knee flexor strength in patients with grade 2 or higher. Another study with grade 2 and above patients found that the isometric torque of the hamstring muscle group in G patients was weaker compared to healthy controls [28]. The hamstring muscles are not only the primary mechanism of knee flexion but also crucial muscle groups that cushion the joint and protect it from eccentric contraction during the support phase of walking, resulting in limb deceleration. Pathologies such as hamstring tightness can disrupt the body's balance and lead to gait abnormalities [29]. In our study, HL was shorter in G patients, and the regression analysis showed that it significantly negatively affected balance, especially in the R-An, R-Pm, L-An, L-Pm, and L-Pl directions.

Ahlback observed an average decrease of 11% in extension strength in CG and grade 1 and 2 G patients. In grade 4 G, they observed a 45% decrease compared to the CG [30]. In our study, we evaluated grade 3 and 4 G patients and did not observe a statistically significant difference in HL despite the muscles being shorter in G patients. We believe that this discrepancy may be due to the differences in factors such as age and BMI among the G patients in our study compared to the literature.

Based on the observations in the light of studies in the literature, the decrease in QA in G patients leading to genu varum and the force loss due to hamstring muscle shortening may result in impaired knee stability, leading to balance deficits. Additionally, impaired balance can lead to a reduction in daily life activities, progressive functional loss, decreased independence in walking, and difficulties in stair climbing [30- 32]. In our study, we found that G patients experienced balance deficits in all directions in both right and left knees due to hamstring muscle shortening and decrease in QA.

Limitations

In this research, only the relationship between QA and balance performance was analyzed. Further studies can investigate the effects of G on mobilization by conducting different functional tests. In our study, there were differences between the two groups in terms of BMI and height. Therefore, future studies that comprehensively examine BMI and height measurements in G patients are needed. One of the most important limitations of our research is that HL was performed with the sit-and-reach test. It is thought that more specific results will be obtained in studies where HL is analyzed by ultrasonographic methods in more suitable laboratory environments. Again, in our study, patients with G were examined cross-sectionally. Longer-term cohort studies will provide a detailed analysis of the reasons for the deterioration of balance performance due to QA and HL.

## Conclusions

HL and QA have a significant role in determining body balance. In our study, we found that QA decreased in G patients, leading to genu varum in these individuals. The hamstring muscle shortening observed in G patients significantly negatively affected balance, especially in the R-An, R-Pm, L-An, L-Pm, and L-Pl directions. For healthcare professionals preparing treatment protocols for G patients, we recommend providing exercises to improve balance, especially in these directions. Although HL did not differ according to CG in G patients, it was found to play an important role in determining balance performance. It is thought that studies to increase hamstring muscle length in the rehabilitation of patients with G will also increase balance performance. Additionally, our study revealed that G patients had a higher BMI compared to the CG, highlighting the negative impact of excess weight on the knee. In this context, we think that the results of our research will be a reference for the health personnel dealing with these patient groups.
